# Astaxanthin Provides Antioxidant Protection in LPS-Induced Dendritic Cells for Inflammatory Control

**DOI:** 10.3390/md19100534

**Published:** 2021-09-23

**Authors:** Yinyan Yin, Nuo Xu, Tao Qin, Bangyue Zhou, Yi Shi, Xinyi Zhao, Bixia Ma, Zhengzhong Xu, Chunmei Li

**Affiliations:** 1 College of Medicine, Yangzhou University, Yangzhou 225009, China; kf56xunuo58@126.com (N.X.); zby18252737828@163.com (B.Z.); sy15365888238@163.com (Y.S.); 2 Jiangsu Key Laboratory of Experimental and Translational Non-Coding RNA Research, Yangzhou University, Yangzhou 225009, China; 3 College of Veterinary Medicine, Yangzhou University, Yangzhou 225009, China; qintao@yzu.edu.cn (T.Q.); yangzhoudaxuezxy@163.com (X.Z.); 4 College of Food Science and Engineering, Yangzhou University, Yangzhou 225009, China; mabixia@vazyme.com; 5 Jiangsu Key Laboratory of Zoonosis, Yangzhou University, Yangzhou 225009, China; zzxu@yzu.edu.cn

**Keywords:** astaxanthin, oxidative stress, sepsis, dendritic cells, inflammation

## Abstract

Astaxanthin, originating from marine organisms, is a natural bioactive compound with powerful antioxidant activity. Here, we evaluated the antioxidant ability of astaxanthin on dendritic cells (DCs), a key target of immune regulation, for inflammatory control in a sepsis model. Our results showed that astaxanthin suppressed nitric oxide (NO) production, reactive oxygen species (ROS) production, and lipid peroxidation activities in LPS-induced DCs and LPS-challenged mice. Moreover, the reduced glutathione (GSH) levels and the GSH/GSSG ratio were increased, suggesting that astaxanthin elevated the level of cellular reductive status. Meanwhile, the activities of antioxidant enzymes, including glutathione peroxidase (GPx), catalase (CAT), and superoxide dismutase (SOD), were significantly upregulated. Astaxanthin also inhibited the LPS-induced secretions of IL-1β, IL-17, and TGF-β cytokines. Finally, we found that the expressions of heme oxygenase 1 (HO-1) and nuclear factor erythroid 2-related factor 2 (Nrf2) were significantly upregulated by astaxanthin in LPS-induced DCs, suggesting that the HO-1/Nrf2 pathway plays a significant role in the suppression of oxidative stress. These results suggested that astaxanthin possesses strong antioxidant characteristics in DC-related inflammatory responses, which is expected to have potential as a method of sepsis treatment.

## 1. Introduction

Sepsis is an organic dysfunction caused by a disordered host response to infection by viruses, fungi, and bacteria [1,2,3,4], which remains a major cause of morbidity and mortality worldwide, with increased burden in low- and middle-resource settings [5]. In the United States, the treatment of sepsis accounted for more than USD 20 billion (5.2%) in total hospital expenses in 2011 [6]. An extrapolation from high-income country data suggests that on a yearly basis, there are an estimated 31.5 million sepsis and 19.4 million severe sepsis cases, with a potential 5.3 million deaths globally [7]. Although more than 100 clinical therapeutic trials have been conducted, no treatment options for sepsis are currently approved by the US Food and Drug Administration (FDA) [8].

After infection, the components of the pathogen, such as lipopolysaccharide (LPS), a key component of the bacterial cell wall, are recognized by macrophages, dendritic cells (DCs), and other immune cells, and then the overloaded inflammatory immune response is activated in early septic patients [9]. Historically, direct anti-hyperinflammatory strategies that attempt to block cytokines, such as interleukin-1 (IL-1) and tumor necrosis factor (TNF), have been the main therapeutic pathway against sepsis. However, the outcome of life-threatening infection is determined by the endogenous complicated inflammatory response. Therefore, using a conventional anti-inflammatory strategy in sepsis cases has to date failed to improve outcomes [8]. Notably, exposure to LPS can induce the rapid and robust production of reactive oxygen species (ROS), which is also a critical pathological feature in septic patients [10]. Oxidative stress is induced by an imbalanced redox state, involving either the excessive generation of ROS or dysfunction of the antioxidant system [11], resulting in the induction of cellular damage, impairment of the DNA repair system, and mitochondrial dysfunction [12]. A growing number of studies agree that interdependence and interconnection are not to be neglected between oxidative stress and inflammation, which co-exist in the inflamed microenvironment. Abundant ROS are released by inflammatory cells at the inflammatory site, which results in exaggerated oxidative injury. Meanwhile, a large amount of ROS and oxidative stress products strengthen proinflammatory responses [13]. Therefore, versatile antioxidants need to be developed to help control overwhelming oxidative stress and hyperinflammatory responses. 

**Figure 1:**
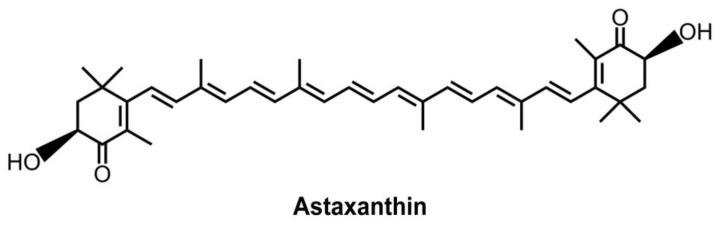
Chemical structure of astaxanthin.

DCs are key regulators of innate and adaptive immunity [14]. The maturation of DCs is directed by signal transduction events downstream of Toll-like receptors (TLRs) and other pattern recognition receptors, following an increase in the production of cytokines, chemokines, and costimulatory molecules [15]. Just as importantly, DCs that possess strong antioxidant systems not only regulate the balance of oxidative stress but also influence the levels of inflammatory responses through the polarization of T cells. Therefore, DCs are an ideal target to manage both oxidative stress and inflammatory responses by some multifunctional antioxidants. Astaxanthin originates from seafood, such as microalgae, trout, yeasts, salmon, and krill [16,17]. Of note, a freshwater unicellular alga, named *Haematococcus pluvialis* (*H. pluvialis*), contains abundant natural astaxanthin [17,18]. Its structure is a xanthophyll carotenoid with hydroxyl and keto moieties on both ends (Figure 1) [19], which effectively scavenges free radicals, thereby protecting fatty acid and biological membranes from oxidative damage [20]. Astaxanthin also can attenuate inflammatory injury caused by diabetes-induced sickness and urate crystal-induced arthritis [21,22]. 

Here, the antioxidant ability of astaxanthin was systematically evaluated on DCs for inflammatory control, which provides evidence that a DC-targeting strategy could be effectively applied in sepsis treatment.

## 2. Results

### 2.1. Astaxanthin Suppressed NO Production in LPS-Induced DCs and LPS-Challenged Mice

**Figure 2:**
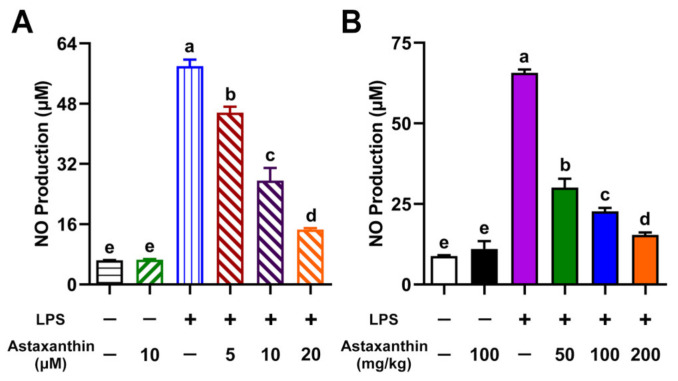
Astaxanthin suppressed the NO production in LPS-induced DCs and LPS-challenged mice. (**A**) DCs were incubated with the indicated concentrations of astaxanthin and LPS (100 ng/mL) for 24 h. (**B**) C57BL/6 mice were orally given astaxanthin before LPS injection. NO production in DC supernatants and serum was detected using the Griess reagent. Results are from one representative experiment of three performed. All of the data are presented as means ± SD. The comparisons were performed with analysis of variance (ANOVA) (multiple groups). Different lowercase letters indicate significant differences between groups (*p* < 0.05).

Nitric oxide (NO) plays a significant role in killing pathogens; however, excessive NO production has been identified as a key pathogenic factor in most immune-mediated diseases [23]. As shown in Figure 2A, LPS was shown to strongly stimulate NO production in DCs compared with an untreated group. Of note, astaxanthin was shown to remarkably suppress NO production in LPS-induced DCs. Many studies have documented an increase in NO production in response to severe sepsis or LPS administration [24]. Therefore, we further examined whether astaxanthin could affect NO levels in LPS-challenged mice. Mice were pre-treated with astaxanthin for 2 days and then injected with LPS. After LPS injection for 4 h, serum samples were collected for NO detection. We found that the administration of astaxanthin significantly decreased NO production in serum after LPS challenge (Figure 2B). Collectively, these findings suggested that astaxanthin strongly inhibited NO production in LPS-induced DCs and LPS-challenged mice.

### 2.2. Astaxanthin Decreased ROS Levels in LPS-Induced DCs

**Figure 3:**
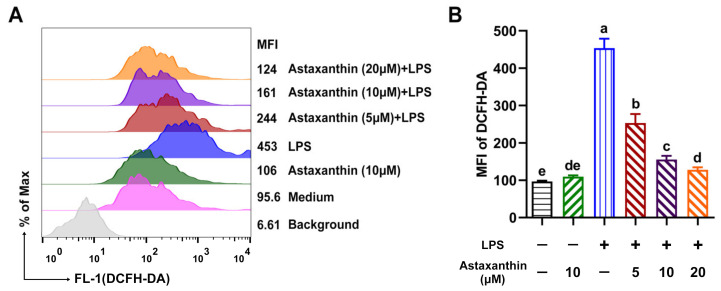
Astaxanthin suppressed the ROS production in LPS-induced DCs. (**A**) After stimulation for 24 h with astaxanthin and LPS (100 ng/mL), DCs were stained with 2′,7′ dichlorofluorescein diacetate (DCFH-DA) and analyzed by flow cytometry (FCM) for ROS detection. (**B**) Results are from one representative experiment of three performed. All of the data are presented as means ± SD. The comparisons were performed with analysis of variance (ANOVA) (multiple groups). Different lowercase letters indicate significant differences between groups (*p* < 0.05).

Oxidative stress refers to elevated intracellular levels of ROS, which result in damage to cellular lipids, proteins, and DNA. Next, intracellular ROS was measured as described previously, with some modifications [25]. As shown in Figure 3, ROS levels were remarkably increased after exposure to LPS for 24 h, whereas astaxanthin strongly attenuated the LPS-induced ROS production in a dose-dependent manner.

### 2.3. Astaxanthin Exhibited Anti-Lipid Peroxidation Activities in LPS-Induced DCs and LPS-Challenged Mice

**Figure 4:**
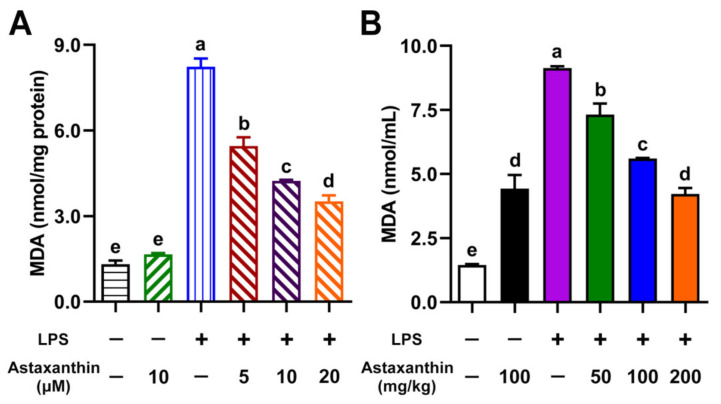
Astaxanthin suppressed lipid peroxidation in LPS-induced DCs and LPS-challenged mice. (**A**) DCs were incubated with the indicated concentrations of astaxanthin and LPS (100 ng/mL) for 24 h. (**B**) C57BL/6 mice were orally given astaxanthin before LPS injection. Blood was sampled at 4 h after LPS injection. The MDA contents in DC lysate supernatants and murine serum were measured as described in the Materials and Methods section. Results are from one representative experiment of three performed. The comparisons were performed with analysis of variance (ANOVA) (multiple groups). Different lowercase letters indicate significant differences between groups (*p* < 0.05).

Maleic dialdehyde (MDA) is commonly known as a marker of oxidative stress and antioxidant status in cells [26]. To investigate whether astaxanthin modulated lipid peroxidation activities in LPS-induced DCs, the intracellular level of MDA was measured. Compared with the control group, the MDA level was significantly elevated in the LPS-only group, while it was remarkably inhibited by the treatment of astaxanthin in a dose-dependent manner (Figure 4A). The serum MDA is a marker of lipid peroxidation in sepsis [27]. Our murine serum results showed a significant decrease in MDA levels after the administration of astaxanthin in LPS-challenged mice (Figure 4B).

### 2.4. Astaxanthin Exhibited Modulating Effects on Intracellular GSH, GSSG, and the GSH/GSSG Ratio in LPS-Induced DCs

**Figure 5:**
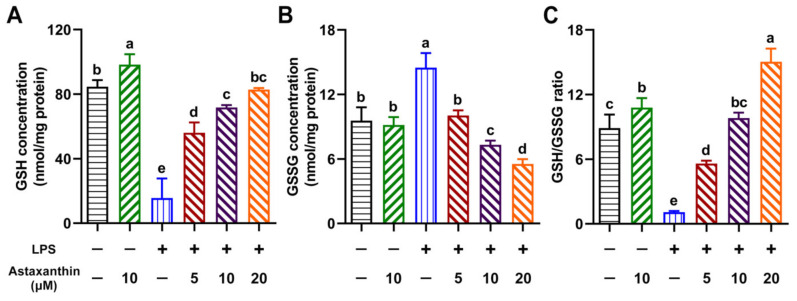
Astaxanthin modulated the intracellular GSH, GSSG, and GSH/GSSG ratio in LPS-induced DCs. After stimulation for 24 h with astaxanthin and LPS (100 ng/mL), the levels of GSH (**A**) and GSSG (**B**), and the ratio of GSH/GSSG (**C**), in DCs were measured as described in the Materials and Methods section. Results are from one representative experiment of three performed. The comparisons were performed with analysis of variance (ANOVA) (multiple groups). Different lowercase letters indicate significant differences between groups (*p* < 0.05).

We further investigated the effects of astaxanthin on the cellular levels of reduced glutathione (GSH), oxidized glutathione (GSSG), and their ratio (GSH/GSSG) in LPS-induced DCs. As shown in Figure 5, LPS significantly decreased the GSH level, increased the GSSG level, and reduced the GSH/GSSG ratio compared with the control. However, astaxanthin remarkably reversed this process in a dose-dependent manner.

### 2.5. Astaxanthin Exhibited Enhancing Effects on Antioxidant Enzyme Activities in LPS-Induced DCs and LPS-Challenged Mice

**Figure 6:**
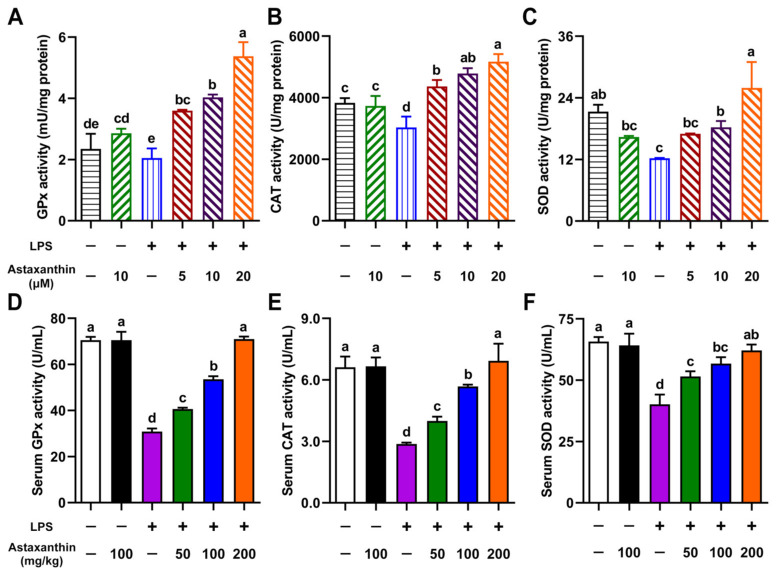
Astaxanthin enhanced the activities of antioxidant enzymes in LPS-induced DCs and LPS-challenged mice. (**A**–**C**) DCs were incubated with the indicated concentrations of astaxanthin and LPS (100 ng/mL) for 24 h. (**D**–**F**) C57BL/6 mice were orally given astaxanthin before LPS injection. Serum was sampled at 4 h after LPS injection. The levels of GPx, CAT, and SOD in the lysate of DCs or serum were measured as described in the Materials and Methods section. Results are from one representative experiment of three performed. Data are presented as means ± SD. The comparisons were performed with analysis of variance (ANOVA) (multiple groups). Different lowercase letters indicate significant differences between groups (*p* < 0.05).

The cells are equipped with a variety of antioxidants, such as glutathione peroxidase (GPx), catalase (CAT), and superoxide dismutase (SOD), which served to counterbalance the effect of oxidants [28]. Therefore, we evaluated the effects of astaxanthin on the activities of antioxidative enzymes (GPx, CAT, and SOD) in LPS-induced DCs. As shown in Figure 6A–C, LPS destroyed the antioxidant system of DCs through decreases in GPx, CAT, and SOD activity. Surprisingly, astaxanthin possessed the ability to increase the activities of these antioxidative enzymes in a dose-dependent manner. Meanwhile, the activities of serum GPx, CAT, and SOD were also detected in LPS-challenged mice. As expected, the administration of astaxanthin remarkably upregulated the serum GPx, CAT, and SOD activities in LPS-challenged mice (Figure 6D–F). Collectively, these results indicated that astaxanthin strongly elevated the activities of the antioxidant enzymes in LPS-induced DCs and LPS-challenged mice.

### 2.6. Astaxanthin Exhibited Inhibitive Effects on Cytokine Production in LPS-Induced DCs and LPS-Challenged Mice

**Figure 7:**
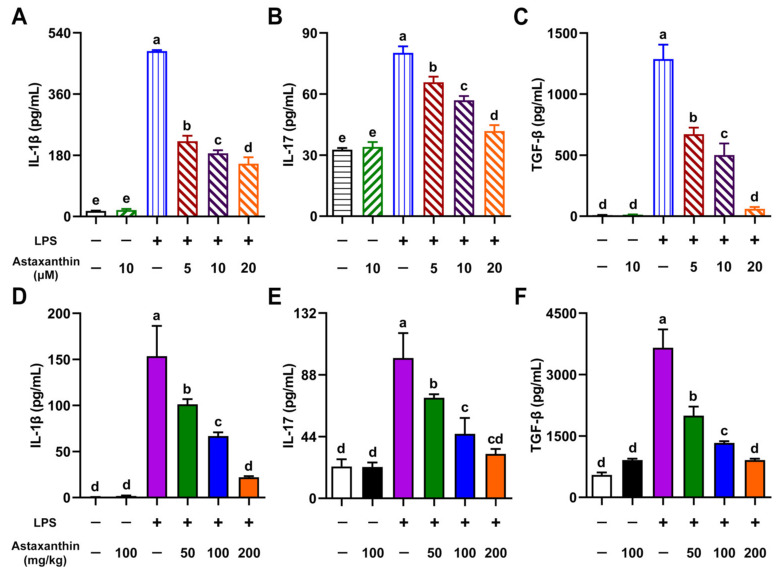
Astaxanthin efficiently impaired the secretion of cytokines in LPS-induced DCs and LPS-challenged mice. (**A**–**C**) DCs were incubated with the indicated concentrations of astaxanthin and LPS (100 ng/mL) for 24 h. (**D**–**F**) C57BL/6 mice were orally given astaxanthin before LPS injection, and then serum was sampled at 4 h after LPS injection. Levels of IL-1β, IL-17, and TGF-β in DC supernatants or serum were measured by ELISA. Results are from one representative experiment of three performed. Data are presented as means ± SD. The comparisons were performed with analysis of variance (ANOVA) (multiple groups). Different lowercase letters indicate significant differences between groups (*p* < 0.05).

To investigate whether astaxanthin modulated the production of cytokines in LPS-induced DCs, the levels of interleukin-1β (IL-1β), interleukin-17 (IL-17), and transforming growth factor-beta (TGF-β) in supernatants of DCs were measured by an enzyme-linked immunosorbent assay (ELISA). After being exposed to LPS (100 ng/mL) for 24 h, the secretion of IL-1β, IL-17, and TGF-β cytokines was upregulated, whereas it was significantly inhibited by the treatment of astaxanthin in a dose-dependent manner (Figure 7A–C). LPS-induced sepsis is associated with overloaded cytokines [29]; therefore, serum samples were collected from mice. As shown in Figure 7D–F, LPS administration markedly increased the levels of IL-1β, IL-17, and TGF-β in mice. As expected, astaxanthin treatment significantly decreased the expression of these cytokines in a dose-dependent manner. These results suggested that astaxanthin strongly inhibited cytokine production in LPS-induced DCs and LPS-challenged mice.

### 2.7. HO-1/Nrf2 Axis Played a Key Role in Suppression of Oxidative Stress in LPS-Induced DCs

**Figure 8:**
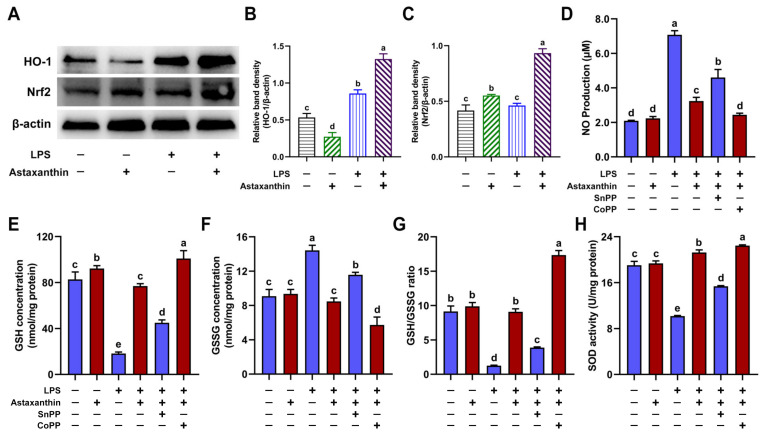
Astaxanthin suppressed oxidative stress via HO-1/Nrf2 axis in LPS-induced DCs. (**A**–**C**) After stimulation for 24 h with astaxanthin and LPS (100 ng/mL), HO-1 and Nrf2 levels were assessed by Western blot. (**D**–**H**) DCs were incubated with astaxanthin (10 μM) and LPS (100 ng/mL) in the presence or absence of SnPP (25 μM) or CoPP (50 μM) for 24 h. (**D**) NO production in DC supernatants was measured using the Griess reagent. (**E**–**H**) The levels of GSH (**E**), GSSG (**F**), and SOD (**H**), and the ratio of GSH/GSSG (**G**), in DCs were measured as described in the Materials and Methods section. Results are from one representative experiment of three performed. Data are presented as means ± SD. The comparisons were performed with analysis of variance (ANOVA) (multiple groups). Different lowercase letters indicate significant differences between groups (*p* < 0.05).

Heme oxygenase 1 (HO-1) and its products can also provide beneficial protection against oxidative injury. Nuclear factor erythroid 2-related factor 2 (Nrf2) is a cytoprotective factor that regulates gene expression for antioxidant and anti-inflammatory properties [30]. Therefore, we detected the expression levels of HO-1 and Nrf2 in DCs by Western blot. Our results demonstrated that astaxanthin treatment significantly upregulated the expression of HO-1 and Nrf2 in LPS-induced DCs (Figure 8A–C). We further investigated whether HO-1 played a significant role in the antioxidant effects of astaxanthin in LPS-induced DCs. We detected the NO production (Figure 8D), intracellular GSH (Figure 8E), GSSG (Figure 8F), the GSH/GSSG ratio (Figure 8G), and the SOD activity (Figure 8H). Significantly, tin protoporphyrin IX (SnPP, an inhibitor of HO-1) reversed the antioxidant effects of astaxanthin in LPS-induced DCs (Figure 8D–H), whereas the suppressive effect of astaxanthin was further aggravated by cobalt protoporphyrin (CoPP, an inducer of HO-1) (Figure 8D–H). Taken together, this showed that the HO-1/Nrf2 axis played a key role in the suppression of oxidative stress in LPS-induced DCs.

## 3. Discussion

**Figure 9:**
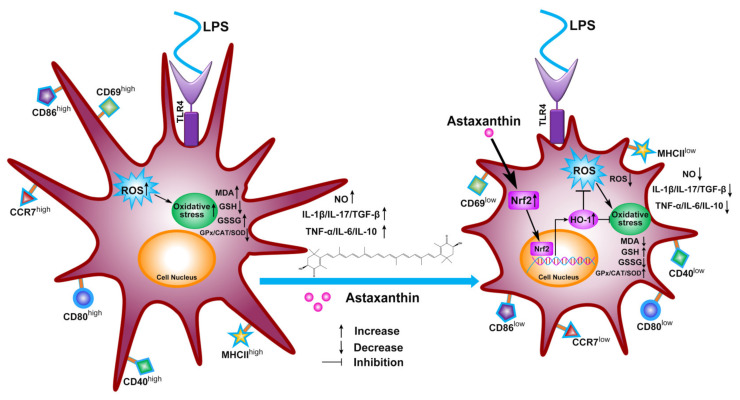
Schematic of proposed mechanism of antioxidant protection of astaxanthin for inflammatory control in LPS-induced DCs. The HO-1/Nrf2 axis was activated by astaxanthin, which inhibited the oxidative stress of LPS-induced DCs, including NO production, ROS production, the lipid peroxidation activities, the GSH level, the GSH/GSSG ratio, and antioxidant enzyme (GPx, CAT, and SOD) activities. These antioxidant properties are conducive to inflammatory controls in DCs, including decreases in levels of activation marker (CD69), the release of cytokines (IL-1β, IL-17, TGF-β, TNF-α, IL-6, and IL-10), phenotypic markers (MHCII, CD40, CD80, and CD86), and a migration marker (CCR7) by astaxanthin.

Previously, we found that astaxanthin strongly inhibited the immune dysfunction of DCs induced by LPS [31]. Here, our work further shows the antioxidative effects of astaxanthin in DCs and mice, which is a potential key aspect of inflammatory control in the sepsis model (Figure 9). These investigational results demonstrated that astaxanthin reduced NO production, ROS production, and lipid peroxidation activities in LPS-induced DCs and LPS-challenged mice. Meanwhile, the GSH level, the GSH/GSSG ratio, and antioxidant enzyme (GPx, CAT, and SOD) activities were upregulated during the above processes. Based on these antioxidant properties, astaxanthin strongly inhibited the cytokine production (IL-1β, IL-17, and TGF-β) in LPS-induced DCs and LPS-challenged mice. Furthermore, we found that the antioxidation mechanism of astaxanthin depended on the HO-1/Nrf2 axis.

NO, an intracellular messenger, regulates cellular functions, such as inflammation and pathogen elimination [32]. However, excess NO can combine with O_2_^−^ to form ONOO^−^, which results in oxidative stress and cellular injury [33]. ROS, generated through a variety of extracellular and intracellular actions, have gained attention as novel signal mediators which are involved in growth, differentiation, progression, and cell death [34]. However, the overproduction of ROS induces significant oxidative stress, resulting in the damage of cell structures, including lipids, membranes, proteins, and DNA [35]. Lipid peroxidation can directly affect the biophysical properties and alter other biophysical characteristics of cell membranes. In addition, cell membrane fluidity is decreased by lipid peroxidation [36]. Meanwhile, ROS can react with polyunsaturated fatty acids of lipid membranes and induce lipid peroxidation [37]. In this study, our results suggested that astaxanthin exerts powerful suppressive effects on NO production, ROS levels, and lipid peroxidation in vitro and in vivo, which play a key role in reversing overloaded LPS-induced oxidative stress.

Previous studies have found that high concentrations of glutathione within cells provide protection against different ROS [32]. GSH, a ubiquitous tripeptide thiol, is known as a vital intracellular and extracellular protective antioxidant, which plays a series of key roles in the control of signaling processes, detoxifying certain xenobiotics and heavy metals [38]. Furthermore, GSH is considered to be one of the most important scavengers of ROS, and its ratio with GSSG may be used as a marker of oxidative stress [38]. The GSH/GSSG redox couple can readily interact with most of the physiologically relevant redox couples, undergoing reversible oxidation or reduction reactions, thereby maintaining the appropriate redox balance in the cells [39]. Under oxidative stress conditions, the GSH can convert itself to GSSG, and the reduction of H_2_O_2_ is catalyzed by the GPx enzyme [40]. Importantly, the addition of astaxanthin to DCs was shown to dramatically attenuate intracellular oxidative stress, indicative of an increase in GSH levels, the GSH/GSSG ratio, and GPx enzyme activity. 

Apart from the GPx, other antioxidant enzymes, including CAT and SOD, also play a very important role in the defense of cells against oxygen-derived free radicals. CAT is a ubiquitous enzyme found in all known organisms, and can transform two H_2_O_2_ into two H_2_O and O_2_ [41]. SOD activity was discovered by McCord and Fridovich in 1969, which can dismutate two superoxide anions (O_2_^−^) into H_2_O_2_ and O_2_ [42]. Our results indicated that astaxanthin significantly upregulates the activities of CAT and SOD, suggesting that the increase in antioxidative enzyme activity might be beneficial to the suppression of oxidative stress.

LPS, derived from Gram-negative bacteria, interacts with Toll-like receptor 4 (TLR4) to cause phagocytic cells to robustly generate a variety of proinflammatory cytokines [43]. Interleukin-1β (IL-1β) is a key proinflammatory cytokine involved in host responses to pathogens and tissue injury [44]. Monocytes, macrophages, and DCs are major IL-1β sources and release this cytokine in response to stimuli such as pathogen-associated or danger-associated molecular patterns (PAMPs or DAMPs) mediated by signaling via several TLR pathways [45]. IL-17 is not only a proinflammatory cytokine, but also a potent mediator of inflammatory responses in various tissues [46]. IL-17 induces multiple genes associated with inflammation, including interleukin-6 (IL-6), and granulocyte-macrophage colony-stimulating factor (GM-CSF) [47,48,49]. In addition, IL-17 enhances the proinflammatory responses induced by IL-1β [50,51], implying that astaxanthin might downregulate the production of IL-1β and IL-17 to protect LPS-induced sepsis. TGF-β is required for IL-17 to produce T helper cell (Th-17 cell) differentiation [52]. In our data, astaxanthin reduced the production of IL-1β, IL-17, and TGF-β in LPS-induced DCs and in LPS-challenged mice, which is in line with our previous findings that showed a decrease in TNF-α, IL-6, and IL-10 caused by astaxanthin in an LPS-induced DC model [31]. These data suggest that astaxanthin, as an antioxidant, can effectively mitigate overloaded cytokine production in vitro and in vivo. The TLR family of receptors can activate the innate immune system by DAMPs that are released during conditions of oxidative stress [53]. ROS from NADPH oxidase can signal the commencement of inflammatory pathways through TLRs. Therefore, we speculated that astaxanthin utilizes its antioxidant property to control inflammation, which might be a promising strategy for treating sepsis. However, the mechanism needs to be further investigated.

Previously, astaxanthin was shown to suppress an LPS-induced increase in inflammatory factors via mitogen-activated protein kinase (MAPK) phosphorylation and nuclear factor-κB (NF-κB) activation in vivo [54]. Here, we demonstrated that astaxanthin inhibited the oxidative stress in LPS-induced DCs and LPS-challenged mice via the activation of the HO-1/Nrf2 pathway. Nrf2 is a transcription factor responsible for the regulation of cellular redox balance and protective antioxidant and phase II detoxification responses [55,56]. Several studies have demonstrated that HO-1 genes are regulated through Nrf2 and play a crucial role in the development of oxidative stress [57]. HO-1, a stress-inducible enzyme, cooperates with NADPH cytochrome P450 to degrade heme in order to produce three bioactive products: iron ions, carbon monoxide (CO), and biliverdin, with the latter being rapidly converted to bilirubin. Biliverdin and bilirubin are potent antioxidants; meanwhile, the other products of HO-1 activity regulate inflammation, apoptosis, and angiogenesis [30]. In addition, CO, an end product of HO-1, can also inhibit NO production and inducible nitric oxide synthase (iNOS) expression via the inactivation of NF-κB [58]. It has been reported that the activation of Nrf2 may prevent an increase in ROS generation through NADPH oxidase [59]. Additionally, the overexpression of HO-1 was also able to inhibit NO production and iNOS expression [58]. Therefore, the activation of the Nrf2/HO-1 axis plays a significant role in protecting host cells against oxidative stress [60].

## 4. Materials and Methods

### 4.1. Ethics Statement

Animal studies were approved by the Jiangsu Administrative Committee for Laboratory Animals (permission number: SYXK(SU)2017-0044) and complied with the guidelines for laboratory animal welfare and ethics of the Jiangsu Administrative Committee for Laboratory Animals.

### 4.2. Materials

Astaxanthin, cobalt protoporphyrin (CoPP, an inducer of HO-1), and LPS (from *Escherichia coli* 026: B6) were obtained from Sigma-Aldrich (St. Louis, MO, USA). Tin protoporphyrin IX (SnPP, an inhibitor of HO-1) was obtained from MedChemExpress (Monmouth Junction, NJ, USA). Rabbit anti-mouse β-actin, rabbit anti-mouse HO-1, and goat anti-rabbit IgG-horseradish peroxidase (HRP) were sourced from Bioworld (St. Louis Park, MN, USA). Recombinant mouse GM-CSF and interleukin-4 (IL-4) were obtained from Peprotech (Rocky Hill, NJ, USA). RPMI 1640 medium and fetal bovine serum (FBS) were sourced from Thermo Fisher Scientific (Waltham, MA, USA). Streptomycin and penicillin were obtained from Invitrogen (Grand Island, NY, USA).

### 4.3. Generation of Bone Marrow-Derived DCs (BMDCs)

BMDCs were isolated and cultured using our improved method [61]. In brief, bone marrow cells were obtained from the tibias and femurs of C57BL/6 mice and cultured in complete medium (RPMI 1640 with 10% FBS, 1% streptomycin and penicillin (Invitrogen, Grand Island, NY, USA), 10 ng/mL GM-CSF and IL-4). The nonadherent cells were discarded and fresh medium was added after 60 h of culture. On day 6, nonadherent and loosely adherent cells were harvested and then cultured overnight. Only cultures with >90% cells expressing CD11c by FCM were used for the experiments.

### 4.4. LPS-Induced Sepsis

C57BL/6 mice, aged 6 weeks old, were randomly divided into five groups (*n* = 10/group), as described previously [31]. Astaxanthin (50, 100, and 200 mg/kg body weight) was orally administered for 2 days every 24 h; 48 h after the 1st oral administration, the mice were injected intraperitoneally with LPS (20 mg/kg body weight). Serum specimens were harvested 4 h after LPS treatment and were stored at −20 °C until use.

### 4.5. Cytokine Assays by ELISA

In vitro, the DCs were incubated with the indicated concentrations of astaxanthin and LPS (100 ng/mL) for 24 h. The culture supernatants were collected. IL-1β, IL-17, and TGF-β in supernatants or serum specimens were determined by using ELISA kits (eBioscience, San Diego, CA, USA) according to the manufacturer’s instructions.

### 4.6. Determination of NO Production

Nitrite (NO_2_^−^) was measured as an indicator of NO synthesis and was estimated using the Griess reagent [62]. A NO detection kit (Beyotime, Shanghai, China) was used according to the manufacturer’s instructions. In brief, 50 μL samples were mixed with an equal volume of Griess reagents I and II at room temperature. The absorbance was determined at 540 nm and calibrated with a nitrite standard curve to determine the nitrate concentration in samples.

### 4.7. Determination of ROS

In brief, after being treated with the indicated concentrations of astaxanthin or astaxanthin plus LPS (100 ng/mL) for 24 h, DCs were collected and incubated with 10 μM DCFH-DA (Beyotime, Shanghai, China) for 20 min at 37 °C. After being washed three times with PBS, ROS generation was analyzed by FCM.

### 4.8. Determination of the Lipid Peroxidation

MDA is the marker of lipid peroxidation [63]. In vitro, after stimulation for 24 h with astaxanthin or astaxanthin plus LPS, DCs were collected and lysed with RIPA buffer. The MDA content in the cell lysate supernatants or serum specimens was measured with thiobarbituric acid (TBA) according to the manufacturer’s instructions (NJBC, Nanjing, China). Briefly, 100 μL samples were mixed with 1 ml of TBA working solution. After being heated for 40 min at 95 °C and cooled to room temperature, the absorbance of the organic layer was determined at 530 nm.

### 4.9. Determination of the GSH and GSSG

In vitro, after stimulation for 24 h with astaxanthin or astaxanthin plus LPS, DCs were lysed by sonication in ice-cold 5% metaphosphoric acid and centrifuged at 10,000× *g* for 20 min to remove debris. The total glutathione (T-GSH) content and GSSG content in the cell lysate supernatants were measured by T-GSH/GSSG kits (NJBC, Nanjing, China) according to the manufacturer’s instructions. The GSH content was obtained by subtracting the 2 × GSSG values from the T-GSH values.

### 4.10. Determination of the Antioxidant Enzyme Activity

In vitro, after stimulation for 24 h with astaxanthin or astaxanthin plus LPS, DCs were collected and lysed with RIPA buffer. The GPx activity in the cell lysate supernatants or serum specimens was measured by the GPx assay kit (NJBC, Nanjing, China). In brief, 10 μL samples were mixed with 10 μL of GPx assay working solution and 176 μL of GPx assay buffer at 25 °C for 5 min. Then, 4 μL of cumene hydroperoxide initiated the reaction and absorbance was measured at 340 nm for 3 min.

The SOD activity in the cell lysate supernatants or serum specimens was measured by the SOD assay kit (NJBC, Nanjing, China) according to the manufacturer’s instructions. The absorbance was measured at 450 nm.

The CAT activity was detected in samples by the CAT assay kit (NJBC, Nanjing, China). Briefly, the samples were treated with excess H_2_O_2_ for an exact time, and a substrate coupled with the remaining H_2_O_2_. After treatment with peroxidase, the absorbance was measured at 520 nm.

### 4.11. Western Blot 

Cells were washed once with ice-cold PBS and lysed with RIPA buffer. Protein concentrations were measured by the bicinchoninic acid (BCA) protein assay kit (Thermo Fisher Scientific, Waltham, MA, USA). Protein extracts were separated on an SDS-PAGE and then transferred to the poly (vinylidene fluoride) (PVDF) membrane. After blocking with 5% dry powdered milk for 2 h, the membrane was immunolabeled with rabbit anti-mouse HO-1 or rabbit anti-mouse β-actin overnight at 4 °C, followed by goat anti-rabbit IgG-HRP for 1 h at room temperature. The membranes were developed in order to visualize the protein by adding an enhanced chemiluminescence reagent (Pierce, Rockford, IL, USA). Autoradiograms were scanned and analyzed with Quantity One (Bio-Rad, Hercules, CA, USA) to quantify band densities. 

### 4.12. Statistical Analysis

The results were expressed as the means ± SD and analyzed with GraphPad Prism 8 software (San Diego, CA, USA). One-way ANOVA analysis of variance was used to compare the variance between different groups. Differences were considered statistically significant when the value of *p* was <0.05.

## 5. Conclusions

In summary, astaxanthin protected LPS-induced DCs and LPS-challenged mice from oxidative stress via the HO-1/Nrf2 axis to achieve overloaded inflammatory control. These data indicate that astaxanthin is a potential candidate drug that could be applied to treat various inflammatory diseases.

## Data Availability

The data presented in this study are available upon request from the corresponding author.
